# shinyExprPortal: a configurable ‘shiny’ portal for sharing analysis of molecular expression data

**DOI:** 10.1093/bioinformatics/btae172

**Published:** 2024-03-29

**Authors:** Rafael Henkin, Katriona Goldmann, Myles Lewis, Michael R Barnes

**Affiliations:** Centre for Translational Bioinformatics, William Harvey Research Institute, Queen Mary University of London, Charterhouse Square, London EC1M 6BQ, United Kingdom; Digital Environment Research Institute, Queen Mary University of London, London E1 1HH, United Kingdom; Centre for Experimental Medicine and Rheumatology, William Harvey Research Institute, Queen Mary University of London, Charterhouse Square, London EC1M 6BQ, United Kingdom; Alan Turing Institute, London NW1 2DB, United Kingdom; Centre for Translational Bioinformatics, William Harvey Research Institute, Queen Mary University of London, Charterhouse Square, London EC1M 6BQ, United Kingdom; Centre for Experimental Medicine and Rheumatology, William Harvey Research Institute, Queen Mary University of London, Charterhouse Square, London EC1M 6BQ, United Kingdom; Centre for Translational Bioinformatics, William Harvey Research Institute, Queen Mary University of London, Charterhouse Square, London EC1M 6BQ, United Kingdom; Digital Environment Research Institute, Queen Mary University of London, London E1 1HH, United Kingdom

## Abstract

**Motivation:**

The scale of omics research presents many obstacles to full sharing and access to analysis results. Current publication models impose limits on the number of pages and figures, requiring careful preparation and selection of content. At the same time, depositing data in open repositories significantly shifts the burden of access and reproduction to readers, who may include people who are not programmers or analysts.

**Results:**

We introduce shinyExprPortal, an R package that implements omics web portals with minimal coding effort. The portals allow exploration of transcriptomic or proteomic expression data and phenotypes, showcasing results of various types of analysis including differential expression, co-expression and pathways analysis. The integration with bioinformatics workflows enables researchers to focus on their results and share findings using interactive and publication-quality plots.

**Availability and implementation:**

The shinyExprPortal package is available to download and install from CRAN and https://github.com/C4TB/shinyExprPortal.

## 1 Introduction

Bioinformatics researchers have many interactive tools at their disposal to conduct omics analysis without having to learn how to code (Barrett *et al.* 2012, Lonsdale *et al.* 2013, Zhou *et al.* 2019). However, when it comes to sharing the results of those analyses, the traditional publication model still imposes limits on the number of pages and figures, thus requiring the careful preparation of content that will convey the key messages of the paper and capture the attention of readers. Other potentially relevant figures, e.g. showing alternative analyses, are relegated to a supplementary material document or are not shown at all. Researchers can also share materials such as raw data, analysis scripts and other documents in repositories such as figshare (https://figshare.com), Zenodo (European Organization For Nuclear Research and OpenAIRE 2013), and OSF (https://osf.io). Readers who are interested in exploring results or expression data, technically minded or not, are then left with the task of sorting through folders and files to hopefully find what is needed to reproduce an analysis.

For transcriptomics data, repositories such as the Gene Expression Omnibus (GEO) allow users to browse and search data from published papers and conduct relatively simple analysis with R scripts, whilst tools that connect to GEO through an application programming interface (API), such as GEOExplorer (Hunt *et al.* 2022), ease the programming demands from the users. In this case, the original researchers no longer have control over the analysis parameters and presentation of their work, possibly leading to misleading findings. To maintain the researcher’s original analysis framework whilst still allowing user exploration, researchers can use packages such as Shiny (Chang *et al.* 2022) and Streamlit (https://streamlit.io/) to create web applications that effectively serve as interactive supplements or data portals (Lewis *et al.* 2019). Creating high-quality portals, however, requires significant effort, expertise and time, with an ongoing maintenance burden. Depending on the structure and timeline of the projects, the long-term sustainability of software also becomes an issue if there is a lack of funding or even interest from authors.

To address these issues, this paper introduces shinyExprPortal, an R package that enables the deployment of web portals for molecular expression data using a text-based configuration file and requiring minimal coding. The package and resulting portal were designed with two general goals in mind: allowing simple data exploration and showcasing analysis results as the original researchers intended them to be presented. Both goals are supported by the use of interactive and publication-quality plots in modules for the exploration of correlations between expression and phenotype data, such as clinical measures of patients, as well as modules for showcasing differential expression analysis, gene co-expression and regulatory networks, and groups of genes in general. The package, therefore, is compatible with the outputs of typical bioinformatics tools such as limma, edgeR, WGCNA, and ARACNe (Margolin *et al.* 2006, Langfelder and Horvath 2008, Robinson *et al.* 2010, Ritchie *et al.* 2015), as well as any lists of genes produced by pathway analysis tools. In principle, any molecular expression matrix can be accommodated, so the portal can support both transcriptomics, proteomics and potentially metabolomics, although different data types cannot be integrated, due to differing data scales. The modular structure of the package means that it can be used as soon as data is available and new analysis results can be included by different members of a research team. It is also easy to extend the deployed portal with custom modules.

## 2 Design rationale

The design of the package followed the workflow of a typical omic analysis project, starting after the initial preprocessing and quality control steps. It was designed in conversation with researchers across three different projects, based on their workflows and requirements: *minimal programming*, *modularity* and *focus on showcase*. The first principle was translated into the use of a text-based configuration file rather than numerous R functions to set up a portal. The second principle enables portals to be incrementally set up as new results emerge, whilst the third one sets the scope for the package: facilitating the sharing of findings rather than being a tool for bioinformatics analysis workflows.

Visitors of the portal can investigate sample subsets of interest and explore correlations between gene expression and observed measures, such as clinical data from trials (e.g. lab tests), or compare changes in clinical data and gene expression over time, e.g. an important measure goes down along with expression. This helps people who do not conduct the actual analysis to think about their data and formulate hypotheses. The showcase modules address the lack of interactivity in typical supplementary materials and the difficulty of reproducing figures.

## 3 Implementation

The package was developed primarily using the shiny package from the R ecosystem. It also relies on sveral surrounding packages that add or extend functionality to web apps created with shiny. The visualizations are primarily rendered using the Vega-lite library (Satyanarayan *et al.* 2017) via the vegawidget (Lyttle 2023) package, with additional views using the iheatmapr (Schep and Kummerfeld 2017) package. Interaction with tabular displays is done using the DT (Xie *et al.* 2023) package. Additional dependencies are found in the package metadata either on CRAN or GitHub.

### 3.1 Data files

Three main data files are required: an expression matrix with samples in columns and genes (or molecules) in rows, a table with rows containing phenotype data (e.g. blood tests, disease activity assessments) for each subject and a lookup/metadata table with rows for samples and subjects identifiers, also including sample/subject metadata (e.g. cell types, time points, control groups). The expression matrix can contain any kind of numeric value that quantifies expression, from counts to abundance. For simple projects where a subject only has one sample, the lookup table is not required in advance and is automatically created by the package. The package does minimal checks, such as ensuring that there are no samples without a subject and vice-versa, but extended quality control tests must be performed by the study authors, as it would be done when performing any downstream analysis.

### 3.2 Configuration file

The configuration file, based on the YAML data interchange format, is the other primary file that the package depends on to run. It contains all the definitions used to assemble the portal and is the main file that users must edit. The format is based on tab stop indentations and has a human-readable syntax. The package includes a step-by-step interactive function to create a configuration file and a function to create a local example; users can also refer to the complete configuration guide in the package documentation or the configuration file used in the live demo. Once the file is created and edited, the portal can be run with a single line of code that points to its location. As such, the portal can be deployed early in the lifecycle of a project and incremented as it progresses. This setup also enables easy versioning of portal and data as no code needs to be re-run or compiled if the file changes.

**Figure 1.**
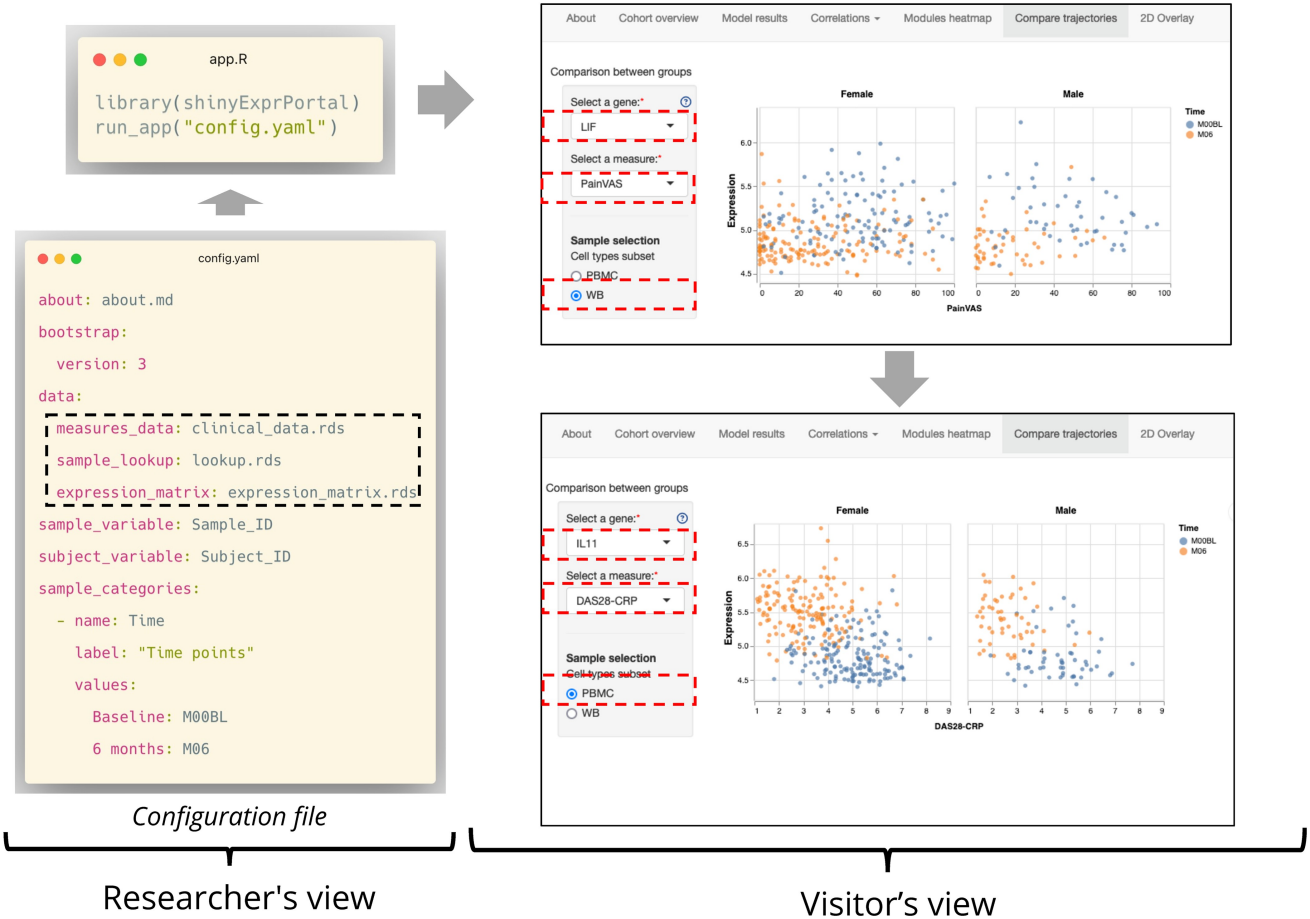
Schematic representation of package use. On the left side, researchers who are setting up the portal create (at least) five files: the expression matrix, the measures (phenotype) data, the lookup table that matches potentially multiple samples to subjects, the configuration file and the app. R file. Visitors accessing the portal can navigate between the modules defined in the configuration file. The example shows a visitor exploring changes between baseline and 6 months, first for one gene, measure and cell type, then switching to another

In a minimal setup, users add portal metadata (name, logo file, landing page text file), information about the expression and measures data and define which modules should appear in the portal. Users must also indicate the columns in their data that identify subjects, samples and metadata for the selection of subsets in the interface. Figure 1 shows an example of the portal metadata followed by the data information.

### 3.3 Data exploration modules

The data exploration modules include two overview modules for time-varying data and three correlation-based modules. These modules do not require additional files beyond the expression matrix and measures data. The cohort overview module shows a grid of small lines, where each line corresponds to a patient’s trajectory for a measure over time (e.g. disease activity). The trajectory comparison between groups module displays scatterplots of expression and an observed measure over time, with one scatterplot per a defined subset of samples. In this view, it is possible to see if changes in expression in a gene are associated with changes in measure over time, and if these changes vary or not between groups of samples. The three correlation modules enable exploring the association between a single gene and various measures, all genes and a single measure and all genes and multiple measures. These associations are displayed through scatterplots, tables and heatmaps, respectively. Portal visitors can choose between different correlation measures (Pearson or Spearman), show or hide a fit line and assign variables from the measures data to colours in the scatterplot.

### 3.4 Analysis showcase modules

The showcase modules include support for differential expression results, heatmaps for groups of genes and precomputed projections of genes with average expression overlays. Some of these modules are directly compatible with the outputs of the packages and tools that are used to compute results, whilst others require additional formatting, e.g. using Excel spreadsheets. The detailed DEG module includes volcano plots to display results, with threshold lines indicating the significance of the genes. This module also includes a table to dig deeper into the results, and it is also used as the default view if a differential expression analysis result is incompatible with a volcano plot. The DEG summary module can be used to show aggregate numbers of differentially expressed genes, with adjusted and unadjusted *P*-values, across multiple models.

Groups of genes extracted from tools such as weighted gene co-expression network analysis (WGCNA), pathways [e.g. KEGG Kanehisa and Goto (2000)] or gene expression signatures can be visualized in the gene modules heatmap, with scatterplots displaying the association between module eigengenes and measures. Finally, the gene projection overlay supports displaying pre-computed projections of genes with annotated clusters and analysing the variation of expression across subsets of samples (e.g. how the expression between clusters varies for a subset of high-disease burden patients).

Each of these modules must have a corresponding section in the configuration file. Some of the fields are required, whilst others can be added for customization (e.g. colours) or to expose or hide certain functions from users (e.g. fit lines in scatterplots). The package documentation includes a complete guide for creating the configuration file, with the required and optional fields as well as examples of how additional files must be created for each module.

### 3.5 Navigation example

Figure 1 illustrates an example of how the package works in practice, as seen in the live demonstration portal (https://c4tb.github.io/ramap_demo/). With the configuration file created and three main data files ready (highlighted by the dashed lines), the portal is set up and a visitor can navigate between the modules. In the figure, the module for comparing changes over time is shown: visitors can choose a gene, a subset of samples and a clinical variable to compare. The interface is populated from the configuration file: the clinical variables that visitors can select are defined by the study authors who are setting up the portal.

### 3.6 Extension and customisation

One of the key aspects of the portal is the support for the addition of new custom modules. By following the examples in the source code, users can extend existing modules and create new ones with additional functionality. Whilst the package has been created with specific goals in mind, this feature allows users to integrate their own plots or analyses, e.g. co-expression networks. As part of the shiny ecosystem, the package also supports the use of Bootswatch themes (https://bootswatch.com/) to change the overall look of the portal. Many of the implemented modules also include options to change palettes in plots to align the portal with figures published in the papers.

## 4 Conclusion

We described an R package that facilitates the sharing of analysis of molecular expression data through interactive web portals. By focusing on the creation of a text-based configuration file, it enables easier deployment and maintenance without significant programming requirements. The package contains a set of core visualization modules but can be extensible by researchers who want to display other kinds of results or plots that are not currently supported.

Unlike current alternatives for sharing findings, such as static documents for supplementary materials and open repositories, shinyExprPortal allows researchers to comment and curate their findings whilst still enabling interested readers to explore alternatives and interact with the results.

## Data Availability

Complete documentation available at https://c4tb.github.io/shinyExprPortal/index.html and live demonstration available at https://c4tb.github.io/ramap_demo/.

## References

[btae172-B1] Barrett T , Wilhite SE, Ledoux P et al NCBI GEO: archive for functional genomics data sets—update. Nucleic Acids Res 2012;41:D991–95. 10.1093/nar/gks1193 23193258 PMC3531084

[btae172-B2] Chang W , Cheng J, Allaire JJ et al *Shiny: Web Application Framework for R*. 2022.

[btae172-B3] European Organization For Nuclear Research and OpenAIRE. *Zenodo: Research. Shared*. 2013. 10.25495/7GXK-RD71

[btae172-B4] Hunt GP , Grassi L, Henkin R et al GEOexplorer: a webserver for gene expression analysis and visualisation. Nucleic Acids Res 2022;50:W367–74. 10.1093/nar/gkac364 35609980 PMC9252785

[btae172-B5] Kanehisa M , Goto S. KEGG: Kyoto Encyclopedia of Genes and Genomes. Nucleic Acids Res 2000;28:27–30. 10.1093/nar/28.1.27 10592173 PMC102409

[btae172-B6] Langfelder P , Horvath S. WGCNA: an R package for weighted correlation network analysis. BMC Bioinformatics 2008;9:559. 10.1186/1471-2105-9-559 19114008 PMC2631488

[btae172-B7] Lewis MJ , Barnes MR, Blighe K et al Molecular portraits of early rheumatoid arthritis identify clinical and treatment response phenotypes. Cell Rep 2019;28:2455–70.e5. 10.1016/j.celrep.2019.07.091 31461658 PMC6718830

[btae172-B8] Lonsdale J , Thomas J, Salvatore M et al The genotype-tissue expression (GTEx) project. Nat Genet 2013;45:580–5. 10.1038/ng.2653 23715323 PMC4010069

[btae172-B9] Lyttle I, Vega/Vega-Lite Developers. *Vegawidget: ’htmlwidget’ for ’Vega’ and ’Vega-Lite’*. 2023.

[btae172-B10] Margolin AA , Nemenman I, Basso K et al ARACNE: an algorithm for the reconstruction of gene regulatory networks in a mammalian cellular context. BMC Bioinformatics 2006;7:S7. 10.1186/1471-2105-7-S1-S7 PMC1810318 16723010

[btae172-B11] Ritchie ME , Phipson B, Wu D et al Limma powers differential expression analyses for RNA-sequencing and microarray studies. Nucleic Acids Res 2015;43:e47. 10.1093/nar/gkv007 25605792 PMC4402510

[btae172-B12] Robinson MD , McCarthy DJ, Smyth GK et al edgeR: a Bioconductor package for differential expression analysis of digital gene expression data. Bioinformatics 2010;26:139–40. 10.1093/bioinformatics/btp616 19910308 PMC2796818

[btae172-B13] Satyanarayan A , Moritz D, Wongsuphasawat K et al Vega-Lite: a grammar of interactive graphics. IEEE Trans Vis Comput Graph 2017;23:341–50. 10.1109/TVCG.2016.2599030 27875150

[btae172-B14] Schep AN , Kummerfeld SK. Iheatmapr: interactive complex heatmaps in R. J Open Source Softw 2017;2:359. 10.21105/joss.00359

[btae172-B15] Xie Y , Cheng J, Tan X et al *DT: A Wrapper of the JavaScript Library ‘DataTables’*. 2023.

[btae172-B16] Zhou Y , Zhou B, Pache L et al Metascape provides a biologist-oriented resource for the analysis of systems-level datasets. Nat Commun 2019;10:1523. 10.1038/s41467-019-09234-6 30944313 PMC6447622

